# Natural variation in photosynthetic capacity, growth, and yield in 64 field-grown wheat genotypes

**DOI:** 10.1093/jxb/eru253

**Published:** 2014-06-24

**Authors:** S. M. Driever, T. Lawson, P. J. Andralojc, C. A. Raines, M. A. J. Parry

**Affiliations:** ^1^School of Biological Sciences, University of Essex, Wivenhoe Park, Colchester, Essex CO4 3SQ, UK; ^2^Plant Biology & Crop Science, Rothamsted Research, West Common, Harpenden, Hertfordshire AL5 2JQ, UK

**Keywords:** Biomass, natural variation, photosynthetic capacity, Rubisco, wheat, yield.

## Abstract

Increasing photosynthesis in wheat has been identified as an approach to enhance crop yield, with manipulation of key genes involved in electron transport and the Calvin cycle as one avenue currently being explored. However, natural variation in photosynthetic capacity is a currently unexploited genetic resource for potential crop improvement. Using gas-exchange analysis and protein analysis, the existing natural variation in photosynthetic capacity in a diverse panel of 64 elite wheat cultivars grown in the field was examined relative to growth traits, including biomass and harvest index. Significant variations in photosynthetic capacity, biomass, and yield were observed, although no consistent correlation was found between photosynthetic capacity of the flag leaf and grain yield when all cultivars were compared. The majority of the variation in photosynthesis could be explained by components related to maximum capacity and operational rates of CO_2_ assimilation, and to CO_2_ diffusion. Cluster analysis revealed that cultivars may have been bred unintentionally for desirable traits at the expense of photosynthetic capacity. These findings suggest that there is significant underutilized photosynthetic capacity among existing wheat varieties. Our observations are discussed in the context of exploiting existing natural variation in physiological processes for the improvement of photosynthesis in wheat.

## Summary

Significant variation in photosynthesis and growth in 64 wheat cultivars was explained by differences in photosynthetic capacity, operation and CO_2_ diffusion. Natural variation in photosynthesis is an underutilized resource for potential crop improvement.

## Introduction

Globally, wheat is one of the most important crops, providing over 20% of the calories consumed by the world’s population and a similar proportion of protein for about 2.5 billion people (Braun *et al.*, 2010). Current increases in global wheat productivity are only 1.1% per annum (Dixon *et al.*, 2009) or even static in some regions (Brisson *et al.*, 2010), while the predicted global demand is likely to increase by 1.7% per annum until 2050 (Rosegrant and Agcaoili, 2010). It is clear that the current yield gain per annum in wheat is insufficient to meet the growing demand, and that new approaches to increasing productivity are essential to avoid shortfalls of growing severity (Hawkesford *et al.*, 2013).

Employing more land for the production of food is not a sustainable option, and thus the productivity of existing arable land will have to be improved. The general consensus is that the only way to improve yield potential in crops, including wheat, is through the improvement of radiation-use efficiency. In the absence of chronic environmental stress, parameters such as harvest index are already close to the theoretical limit (Foulkes *et al.*, 2011; Reynolds *et al.*, 2012a). Photosynthesis appears to be a process for which significant improvement in radiation-use efficiency is still possible, both for wheat (Parry *et al.*, 2011) and for other crop species (Long *et al.*, 2006; Zhu *et al.*, 2010; Raines, 2011). In wheat, evidence for increased yield in response to CO_2_ enrichment (Ainsworth and Long, 2005), and the positive relationship between photosynthesis and biomass (Kruger and Volin, 2006) and yield (Fischer *et al.*, 1998), support this contention. In principle, improved photosynthesis could be achieved by increasing photosynthetic rate per unit leaf area and by optimizing light interception and utilization through modification of canopy architecture and photosynthetic duration.

For wheat, several strategies have been proposed for increasing photosynthetic rate per unit leaf area. As reviewed by Parry *et al.* (2011), these include improvement of ribulose-1,5-bisphosphate carboxylase/oxygenase (Rubisco) activity, faster regeneration of ribulose-1,5-bisphosphate (RuBP), and the introduction of carbon-concentrating mechanisms. These strategies all require modification of the photosynthetic components, which can only be achieved through genetic manipulation. However, although modern techniques allow the extensive manipulations that are necessary for the introduction of complex processes such as a carbon-concentrating mechanism, there is still much to be learned from the natural variation in photosynthetic capacity and performance that already exists between species and within cultivars, as well as their ability to survive or thrive under specific environmental stresses. The physiological or genetic mechanisms that underlie such natural variation in species or cultivars are largely untapped resources that may provide not only valuable information on the capacity and performance of different cultivars under different environmental conditions but also an invaluable genetic resource that can be used to improve yield (Flood *et al.*, 2011; Lawson *et al.*, 2012). Knowledge of this natural diversity will encourage the use of new cultivar backgrounds (with desirable traits) onto which additional genetic modifications can be targeted in a bid to improve crop yields. Work by Wullschleger (1993) was one of the first large-scale studies that showed significant variation in photosynthetic capacity in a range of species. CO_2_ assimilation and intercellular CO_2_ concentration analysis of 109 species (ranging from woody perennials to herbaceous annuals) revealed that species-specific differences in CO_2_ assimilation rate were due to differences in underlying biochemistry including carboxylation capacity and RuBP regeneration (via electron transport).

Despite photosynthesis being the primary determinant of plant productivity, previous research has seldom provided good evidence for a strong positive relationship between the rate of photosynthesis per unit leaf area and yield. Most previous studies assessing variation in wheat have involved limited measurements on small sets of germplasm. For example, past efforts to investigate photosynthetic variation in wheat used up to 48 different genotypes (Fischer *et al*., 1981, 1998; Blum, 1990; Watanabe *et al.*, 1994; Reynolds *et al.*, 2000; Xue *et al.*, 2002; Chytyk *et al.*, 2011; Sadras *et al.*, 2012). However, the information available across these studies is not directly comparable, due to the application of different experimental approaches. For example, some studies assessed photosynthetic characteristics in response to changes in light and CO_2_ concentration (Blum, 1990; Chytyk *et al.*, 2011), while others relied on steady-state measurements taken under a variety of different conditions (Fischer *et al*., 1981, 1998; Watanabe *et al.*, 1994; Reynolds *et al.*, 2000; Xue *et al.*, 2002; Sadras *et al.*, 2012). Therefore, the extent to which photosynthetic characteristics vary in existing wheat cultivars remains unclear.

The aims of this study were to explore the naturally existing variation in photosynthetic characteristics in wheat, to identify any possible correlations with yield. The longer-term aim would be to exploit this information to assist in the identification of targets for the improvement of crop yield. To achieve this, a diverse panel of 64 modern elite wheat cultivars differing in geographical location, year of introduction, and intended end use (feed and bread varieties being represented) were grown in the field and a range of growth, yield, and photosynthetic parameters determined. Analysis of these data has provided an insight in the variation in physiological processes, which are discussed together with the possibilities for improving photosynthesis in wheat.

## Materials and methods

### Plant material, field conditions, and harvest

The 64 wheat cultivars used in this study were from the Earliness & Resilience for Yield in a Changed Climate (ERYCC) panel that were generated as part of a Department for Environment, Food and Rural Affairs and HGCA sponsored sustainable Arable LINK project (‘Adapting Wheat to Global Warming’; Project LK0992) by Clarke *et al.* (2012). Within the ERYCC LINK project, wheat cultivars were selected on the basis of phenology (for example, lodging resistance) and yield (Table 1). Certain cultivars also contained specific yield-related genes, such as *Rht* genes, determining stature and grain number; *Ppd1* and *Ppd2*, photoperiod genes governing floral growth rate and apex morphology; *Lr37* and *Pch1*, conferring resistance to leaf rust and eyespot disease, respectively; *Sm1*, conferring resistance to orange wheat blossom midge; and *1RS*, a rye chromosome arm, which is a source of genes for both insect and disease resistance in wheat (Table 1).

**Table 1.** List of cultivars grown in the field in the current study with specification of common names, indication of landmark variety, year of introduction, indication of bread or feed variety (for landmark varieties), and presence of genes of interestThese cultivars were chosen based on the ERYCC LINK project (‘Adapting wheat to global warming’, Sustainable Arable LINK Programme Project LK0992).

Cultivar	Name	Year of introduction	Landmark variety (Y/N)	Bread/feed variety (Landmark)	Genes
1	Equinox	1997	N	–	*Rht2 Rht8 Lr37 Sm1 1RS*
2	Cordiale	2004	Y	Bread	*Rht2 Rht8 Sm1*
3	Robigus	2003	Y	Feed	*Rht1 Rht8 Sm1*
4	Access	2002	N	–	*Rht2 Rht8 Lr37 Sm1 1RS*
5	Oakley	2007	Y	Feed	*Rht1 Rht8 Sm1*
6	Humber	2007	N	–	*Rht2 Rht8 Lr37 Sm1 1RS*
7	Malacca	1999	Y	Bread	*Rht2 Rht8 Sm1*
8	Dover	2007	N	–	*Rht2 Rht8 Lr37 Sm1*
9	Beaver	1990	Y	Feed	*Rht2 Rht8 Sm1*
10	Andalou	2002	N	–	*Ppd1 Rht2 Rht8 Sm1*
11	Royssac	2003	N	–	*Ppd1 Rht2 Rht8 Sm1*
12	Exotic	2006	N	–	*Ppd1 Rht2 Rht8 Lr37 Sm1*
13	Rialto	1995	Y	Bread	*Rht2 Rht8 Sm1 1RS*
14	Exsept	2001	N	–	*Rht2 Rht8 Sm1*
15	Einstein	2003	Y	Bread	*Rht2 Rht8 Sm1*
16	Ambrosia	2005	N	–	*Rht2 Rht8 Sm1*
17	Claire	1999	Y	Feed	*Rht2 Rht8 Lr37 Sm1 1RS*
18	Glasgow	2005	N	–	*Rht2 Rht8 Sm1*
19	Alchemy	2006	Y	Feed	*Rht2 Rht8 Sm1*
20	Istabraq	2004	N	–	*Rht2 Rht8 Sm1*
21	Soissons	1995	N	–	*Ppd1 Rht1 Rht8 Sm1*
22	Sankara	2005	N	–	*Rht2 Rht8 Lr37 Pch1 Sm1*
23	Mendel	2005	N	–	*Ppd2 Rht2 Rht8 Sm1*
24	Mercato	2006	N	–	*Ppd1 Rht1 Rht8 Lr37 Sm1*
25	Deben	2001	N	–	*Rht2 Rht8 Sm1*
26	Xi19	2002	N	–	*Rht2 Rht8 Sm1*
27	Solstice	2002	Y	Bread	*Rht2 Sm1*
28	Gladiator	2004	N	–	*Rht2 Rht8 Lr37 Sm1 1RS*
29	Brompton	2005	N	–	*Rht2 Rht8 Sm1 1RS*
30	Mascot	2006	N	–	*Rht2 Rht8 Lr37 Sm1*
31	Zebedee	2007	N	–	*Rht2 Rht8 Sm1*
32	Gatsby	2006	N	–	*Rht1 Rht8 Sm1 1RS*
33	Hyperion	2006	N	–	*Rht2 Rht8 Lr37 Pch1 Sm1*
34	Gulliver	2008	N	–	*Rht2 Rht8 Lr37 Sm1*
35	Timber	2007	N	–	*Rht2 Rht8 Sm1*
36	Consort	1995	Y	Feed	*Rht2 Rht8 Sm1*
37	Battalion	2007	N	–	*Rht2 Rht8 Lr37 Pch1 Sm1*
38	Marksman	2008	N	–	*Rht2 Rht8 Lr37 Pch1 Sm1*
39	Musketeer	2008	N	–	*Rht2 Rht8 Lr37 Pch1 Sm1*
40	Recital	1986	N	–	*Ppd1 Ppd2 Rht1 Rht8 Sm1*
41	Hereward	1991	Y	Bread	*Rht2 Rht8 Sm1*
42	Apache	1998	N	–	*Ppd1 Rht8 Lr37 Sm1*
43	Caphorn	2002	N	–	*Ppd1 Rht2 Rht8 Lr37 Sm1*
44	Galahad	1983	Y	Feed	*Rht2 Rht8 Sm1*
45	Alixan	2005	N	–	*Rht2 Rht8 Sm1*
46	Avalon	1980	Y	Bread	*Rht2 Rht8 Sm1*
47	Cappelle Desprez	1953	Y	Feed	*Rht8 Sm1*
48	Haven	1990	N	–	*Rht2 Rht8 Sm1 1RS*
49	Cezanne	1998	N	–	*Ppd1 Rht1 Rht8 Sm1*
50	Savannah	1998	N	–	*Rht2 Rht8 Lr37 Sm1 1RS*
51	Maris Huntsman	1998	N	–	*Rht8 Sm1*
52	Paragon	1999	N	–	*Rht8 Sm1*
53	Riband	1989	Y	Feed	*Rht2 Rht8 Sm1*
54	Norman	1981	Y	Feed	*Rht2 Rht8 Sm1*
55	Buster	1995	N	–	*Rht2 Rht8 Sm1*
56	Spark	1993	N	–	*Rht8 Sm1*
57	Mercia	1986	Y	Bread	*Rht8 Sm1*
58	Cadenza	1994	N	–	*Rht8 Sm1*
59	Hobbit	1977	N	–	*Rht2 Rht8 Sm1*
60	Hustler	1978	N	–	*Rht2 Rht8 Sm1*
61	Longbow	1983	N	–	*Rht2 Rht8 Sm1*
62	Maris Widgeon	1964	Y	Bread	*Rht8 Sm1*
63	Virtue	1979	N	–	*Rht2 Rht8 Sm1*
64	Bacanora	1988	N	–	*Ppd1 Rht1 Rht8 Sm1 1RS*

Within the panel, all cultivars were introduced after 1975, with the exception of cultivars 62 (Maris Widgeon, 1964) and 47 (Capelle Desprez, 1953). Thirty-four of the chosen cultivars have been introduced since 2000 (Supplementary Fig. S1 available at *JXB* online). However, although consisting predominantly of modern varieties, a consistent increase in yield with year of introduction has been observed from the cultivars within this panel (Clarke *et al.*, 2012).

The field in which the material was grown (Great Field 1 and 2) was composed of a moderately well drained flinty loam on clay with flints and/or chalk, which had been used for oilseed rape production in the preceding season. The ground was prepared by application of systemic herbicide, followed 3 weeks later by ploughing, with cultipressing and power harrowing at intervals of 1 week thereafter, to produce a suitable seed bed. Immediately afterwards (5 October 2011), the wheat seed was drilled at a rate of 350 seeds m^–2^ and the plots rolled. Three (2×1 m) plots of each cultivar were arranged in separate, randomized blocks, each block containing eight rows with eight plots per row, and three blocks in total (three blocks of 64 cultivars each=192 plots) with 1.0 m between rows and 0.5 m between adjacent plots. The plots were treated periodically, pre- and post-emergence, with herbicides, insecticides, and fungicides to promote weed- and disease-free development. Nitrogen and sulfur (Doubletop: GrowHow UK) and nitrogen alone (F34 Nitram: GrowHow UK) were applied in mid-March and mid-May, respectively (185kg ha^–1^ on each occasion). The tall cultivars 62 and 47 (Maris Widgeon and Cappelle Desprez) were staked to reduce lodging.

Harvest took place (17 August 2012) once all the cultivars had reached physiological maturity and the kernels were hard (Zadoks scale 9.1–9.2). Grain yields were obtained with a Haldrup plot combine, the straw being weighed on the back of the combine by means of a supplementary load cell. The straw was immediately subsampled, bagged, and chopped for determination of moisture content by oven drying. A fresh grain subsample was also taken for moisture determination.

Development according to the Zadoks scale of all 64 cultivars is shown in Supplementary Fig. S2 available at *JXB* online.

### *A*/*C*_i_ photosynthetic gas-exchange measurements

Photosynthesis measurements were performed on flag leaves that had fully emerged, between the flag leaf sheath extension and boot (sheath) swelling (Zadoks growth stages 4.1–4.5). Measurements were made pre-anthesis to ensure that differences in sink size did not influence photosynthetic capacity. In order to ensure that all genotypes were measured under identical conditions, whole shoots were collected before dawn by cutting the base of the stem, followed by immediate recutting of the shoot (5–10cm above the original incision) under water. Shoots were promptly transferred to the laboratory in tubes containing demineralized water and stored in a controlled environment cabinet providing darkness, low temperature, and high humidity (10 °C and 90% relative humidity), which simulated prevailing night-time conditions. Prior to gas-exchange measurements, while still in darkness, the flag leaf was cut under water at the base of the lamina and the cut base placed in a tube containing demineralized water to a depth of approximately 3cm. It was then transferred to a second controlled environment room and left to acclimate for 1h at 15 °C and 60% relative humidity, with an irradiance [photosynthetic photon flux density (PPFD)] of 500 µmol m^–2^ s^–1^, which was sufficient for light adaptation. Daily mean light levels in the field for the period of photosynthetic measurements are given in Supplementary Table S1 available at *JXB* online.

The response of photosynthesis to changes in *C*_i_ was measured in the middle of the flag leaf with a near-saturating irradiance of 1500 μmol of photons m^−2^ s^−1^, using an open infrared gas-exchange system and a 2cm^2^ leaf chamber with an integral blue–red LED light source (LI-6400–40; LI-COR, Lincoln, NE). Leaves were clamped in the leaf chamber and complete sealing of the gaskets around the leaf was assured to prevent possible diffusion leakage. Leaf temperature was maintained at 20±1 °C with a vapour pressure deficit of 0.9 kPa and an ambient CO_2_ concentration (*C*_a_) of 400 μmol mol^−1^. Subsequently, *C*_a_ was decreased to 300, 200, 100, and 75 μmol mol^−1^ before returning to the initial concentration. This was followed by an increase to 550, 700, 1000, and 1200 μmol mol^−1^. Readings were recorded when CO_2_ assimilation (*A*) had stabilized to the new conditions (after about 2min). The maximum velocity of Rubisco for carboxylation (*V*_cmax_), the maximum rate of electron transport demand for RuBP regeneration (*J*_max_), mesophyll conductance (*g*_m_), and respiration rate (*R*_d_) were derived by curve fitting as described by Sharkey *et al.* (2007) using the Rubisco kinetic constants for wheat (Carmo-Silva *et al.*, 2010). Operational assimilation rate under ambient conditions (*A*_400_) and maximal carboxylation rate (*A*_max_) were determined from assimilation values recorded at 400 and 1200 μmol mol^−1^ CO_2_ concentration, respectively.

### Protein extraction, Rubisco quantification, and carboxylation efficiency

Immediately after measurement of leaf photosynthesis, a 3–4cm leaf sample encompassing the area within the leaf chamber was taken by cutting with a razor blade perpendicular to the central vein. The length and width at both ends of the leaf section were measured, followed by snap freezing in liquid N_2_ and storage at –80 °C, awaiting extraction of soluble leaf protein. These samples (leaf area 6–8cm^2^) were ground with an ice-cold pestle and mortar to a particle-free homogenate (in ≤90 s) *immediately after* addition to 1.5ml of ice-cold buffer, containing 50mM MES/NaOH (pH 7.0), 10mM MgCl_2_, 1mM EDTA, 1mM EGTA, 50mM 2-mercaptoethanol, 2mM benzamidine, 5mM ε-aminocaproic acid, 10mM dithiothreitol, 10mM NaHCO_3_, 1mM PMSF, 1/100vol. plant Protease Inhibitor Cocktail (Sigma), 20mg of insoluble polyvinylpyrrolidone, and 100mg acid-washed sand. (The last six components were added just before extraction, either from concentrated stock solutions or in solid form.) The homogenate was clarified by centrifugation (14 700*g*, 5min, 4 °C). Rubisco in the supernatant was quantified and carboxylation efficiency was determined by a modification of the method of Yokota and Canvin (1985). Duplicate 150 μl aliquots of the supernatant were mixed immediately with 150 μl of [^14^C]2′-carboxyarabinitol-1, 5-bisphosphate (CABP)-binding solution at 0 °C, containing 200mM Bicine/NaOH (pH 8.0), 40mM MgCl_2_, 20mM NaHCO_3_, 100mM 2-mercaptoethanol, 200mM Na_2_SO_4_, and 15 nmol (0.56 kBq) [2’-^14^C]CABP. (The remaining supernatant was snap frozen immediately in liquid N_2_ and stored at –80 °C.) After 20min at 0 °C, 60% (w/v) polyethylene glycol (PEG) 4000 was added with thorough mixing, to give a final concentration of 25% PEG, causing the precipitation of Rubisco and Rubisco-bound [^14^C]CABP. After 30min at 0 °C, the precipitate was sedimented by centrifugation (14 *700g,* 10min, 4 °C), the supernatant discarded, and the pellet washed by repeated vortexing with 500 μl of 20% (w/v) PEG 4000, containing 100mM Bicine/NaOH (pH 8.0), 20mM MgCl_2_, 10mM NaHCO_3_,and 50mM 2-mercaptoethanol. After 15min at 0 °C, the pellet was consolidated by centrifugation (14 700*g*, 10min, 4 °C) and the wash/centrifugation procedure repeated once more. The final pellet was redissolved in 500 μl of 1% (v/v) Triton X-100. The ^14^C content was determined by liquid scintillation counting, after addition to 4ml of scintillation cocktail (Ultima Gold; Perkin Elmer, UK). Total protein content of extracted soluble protein samples was determined according to Bradford (1976).

### Statistical analysis

Correlations between all possible pairs of measured parameters were analysed by Spearman’s rank correlation test (Spearman, 1987), using observations of all cultivars. All parameters were tested for normal distribution using a Shapiro–Wilk test (Shapiro and Wilk, 1965), and for each parameter (or trait), differences between cultivars were determined by analysis of variance (ANOVA). Principal component analysis (PCA) was applied to the cultivar by trait (C×T) matrix of means with standardized data transformation, similar to a genotype by environment (G×E) analysis. Hierarchical clustering was performed using Ward’s minimum variance method (Ward, 1963) with standardized data transformation of the C×T matrix of means and a cut-off of four cluster groups. Differences between clusters were determined with ANOVA and a Siegel–Tukey’s post-hoc rank test (Siegel and Tukey, 1960). Variance and residual variance, determined by restricted maximum-likelihood analysis using a linear mixed model, were used to calculate heritability in the narrow sense (*h* ^2^) according to Nyquist (1991). All analysis was done using R software (R Core Team, 2012).

## Results

**Fig. 1.**
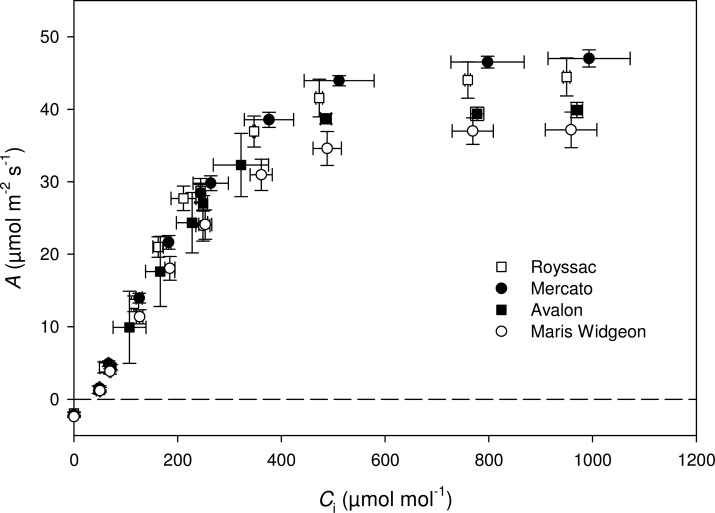
Example of variation observed in data of the response of photosynthetic CO_2_ assimilation (*A*) to different internal CO_2_ concentrations (*C*_i_), or *A*/*C*_i_ curve, for four different cultivars. Means of three replicates with standard deviations are shown.

**Fig. 2.**
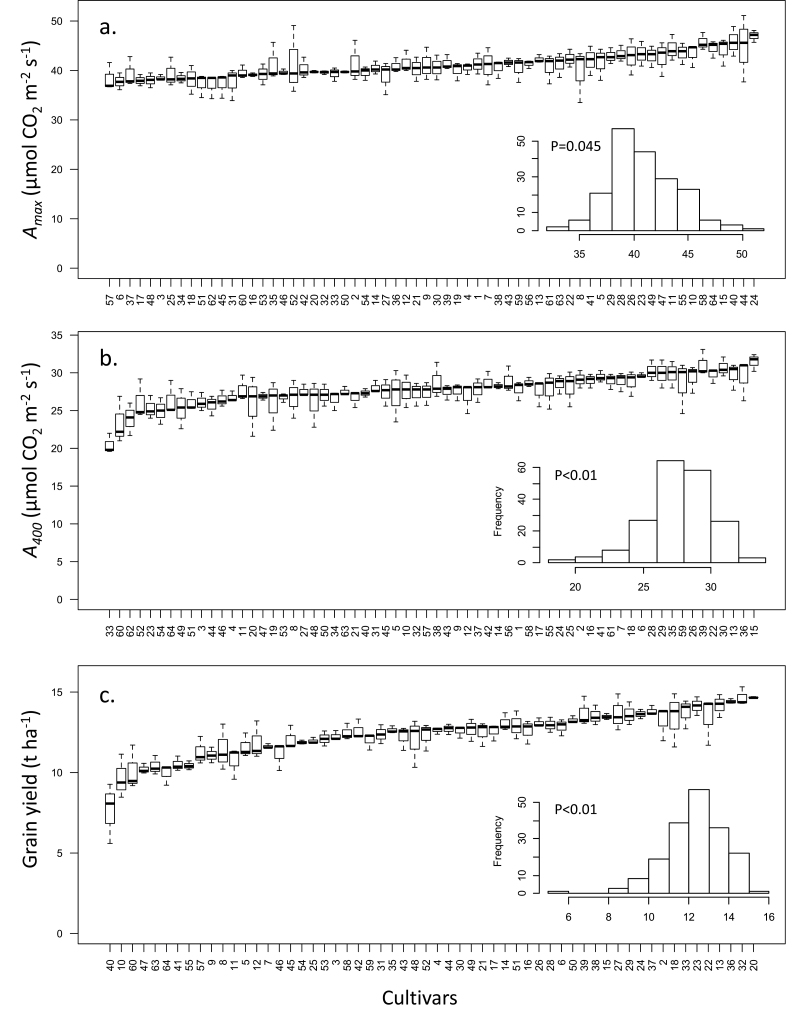
Mean and variation of all cultivars for (a) maximum photosynthetic CO_2_ assimilation (*A*_max_) at saturating CO_2_ concentration, (b) photosynthetic CO_2_ assimilation at ambient CO_2_ concentration (400 µmol mol^–1^ CO_2_, *A*_400_), and (c) grain yield. Cultivars are ranked according to increasing mean of each parameter. Insets show histograms of frequency distribution of respective parameters with *P* values for normal distribution.

The response of photosynthetic rates to increasing CO_2_ concentration under light-saturated conditions (*A*/*C*_i_ response curve) was determined for the 64 cultivars of field-grown wheat. Typical *A*/*C*_i_ response curves of four of these wheat cultivars highlighted the differences in the CO_2_ saturated rate of photosynthesis (*A*_max_; Fig. 1). Significant differences in *A*_max_ illustrated the variation in maximum photosynthetic capacity among cultivars. This variation was significantly greater than the within-cultivar variation, as demonstrated by the tight error bars. The small errors associated with these measurements also demonstrated the robustness of the technique. Cultivar-specific values of *A*_max_ (Fig. 2a) showed significant differences, with mean values ranging from 38.4 µmol m^–2^ s^–1^ for cultivar 57 (Mercia) to 47.0 µmol m^–2^ s^–1^ for cultivar 24 (Mercato). Differences among cultivars were highly significant (*P*<0.01), and the observations of *A*_max_ for the 64 cultivars were normally distributed (*P*=0.045, inset in Fig. 2a). Similar significant variation was observed in *A*_400_ values (*P*<0.01, Fig. 2b), which were also normally distributed (*P*<0.01; inset in Fig. 2b), and provided an indication of the highest operational carbon assimilation performed by these plants at current atmospheric (400 µmol mol^–1^) CO_2_ concentration, at saturating PPFD. Under these conditions, the lowest mean value of 20.5 µmol m^–2^ s^–1^ was found for cultivar 33 (Hyperion) and the highest for cultivar 15 (Einstein) with a mean value of 31.5 µmol m^–2^ s^–1^. However, although the relative variation of *A*_max_ and *A*_400_ between the cultivars was similar, the ranking of cultivars based on their means were different between the operational assimilation rates (*A*_400_, Fig. 2b) and the maximum assimilation rates (*A*_max_, Fig. 2a), suggesting that operational and maximum rates of photosynthesis were influenced by different factors. A comparable degree of variation was also found in the grain yield (Fig. 2c) with mean values ranging between 7.6 and 14.7 t ha^–1^. The values were normally distributed (*P*<0.01, inset in Fig. 2c) and differences among cultivars were highly significant (*P*<0.01).

**Fig. 3.**
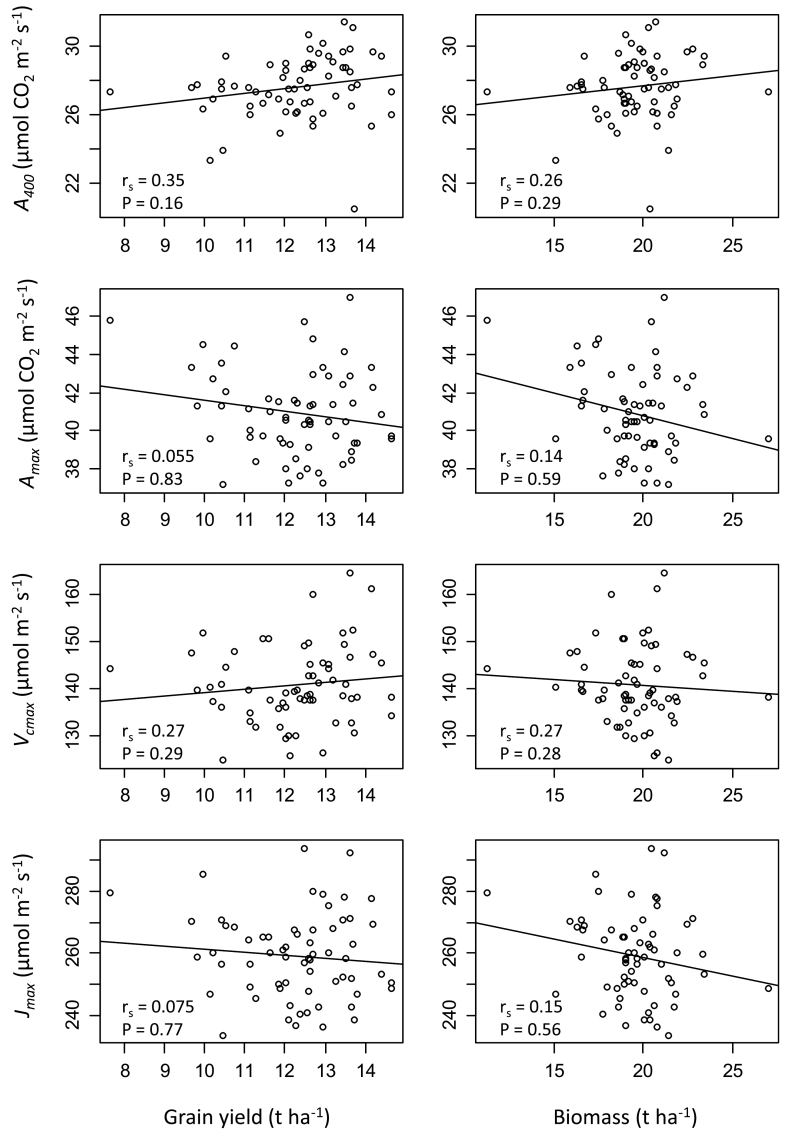
Correlations of photosynthetic parameters operational assimilation rate (*A*_400_), maximum carboxylation rate (*A*_max_), maximum velocity of Rubisco carboxylation (*V*_cmax_) and the maximum rate of electron transport demand for RuBP regeneration (*J*_max_) with grain yield and total aboveground biomass. Correlation (*r*_*s*_), significance (*P* value), and regression line are given for each figure.

It is interesting to note that ranking the cultivars for grain yield from lowest to highest was completely different from the ranking for either of the photosynthetic parameters *A*_max_ and *A*_400_. This suggested little correlation between yield and either *A*_max_ or *A*_400_. In fact, an analysis of selected photosynthetic parameters (*A*_400_, *A*_max_, *V*_cmax_, and *J*_max_) undertaken in this study revealed that no significant correlations could be drawn between photosynthetic parameters of the flag leaf determined on a leaf area basis and either biomass or yield (Fig. 3). A complete correlation analysis including *all* measured parameters showed no significant correlations between photosynthetic parameters and growth or yield-related parameters (Supplementary Fig. S3 available at *JXB* online).

**Fig. 4.**
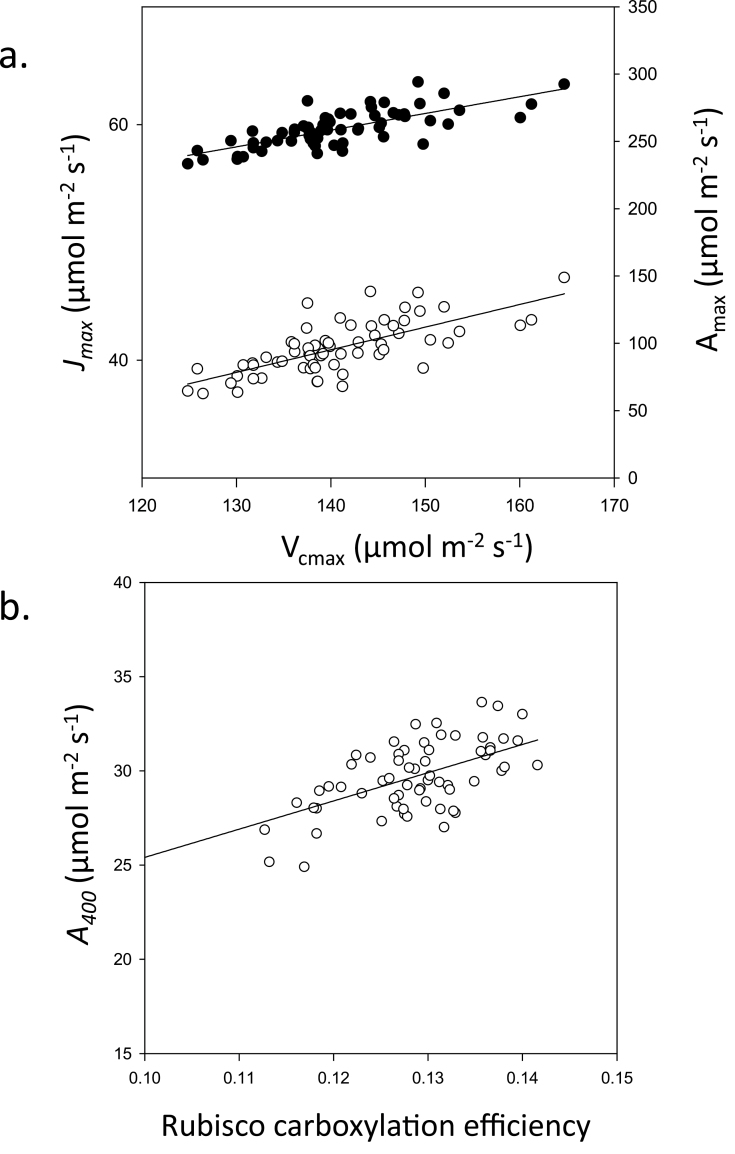
(a) Relationship between the maximum rate of electron transport demand for RuBP regeneration (*J*_max_) and maximum velocity of Rubisco for carboxylation (*V*_cmax_) and the relationship between maximum photosynthetic CO_2_ assimilation at saturating CO_2_ concentration (*A*_max_) and *V*_cmax_. (b) Relationship between the photosynthetic CO_2_ assimilation at ambient CO_2_ concentration (*A*_400_) and the *in vitro* carboxylation efficiency of Rubisco.

The maximum rate of carboxylation by Rubisco (*V*_cmax_) and the maximum electron transport demand for RuBP regeneration (*J*_max_) determined from the *A*/*C*_i_ analysis (Fig. 4a, circles) varied between 124–161 and 233–280 μmol m^–2^ s^–1^, respectively. The lowest *V*_cmax_ values were observed in cultivar 62 (Maris Widgeon) and the highest in cultivar 23 (Mendel). For *J*_max_, the lowest values were observed for cultivar 62 (Maris Widgeon) and highest for cultivar 44 (Galahad). Despite differences among cultivars in parameters such as *A*_max_, *V*_cmax_ and *J*_max_ and the lack of correlation between photosynthetic and measured growth parameters, a strong correlation was nonetheless found between *V*_cmax_ and *J*_max_ (*r*_s_=0.73, *P*<0.01) and between *V*_cmax_ and *A*_max_ (*r*_s_=0.68, *P*<0.01) (Fig. 4a). Carboxylation efficiency calculated from the gradient of the *A*/*C*_i_ curves for *C*_i_ values below 300 ppm (mean gradient *R* ^2^=0.979) also showed a significant correlation with *A*_400_ values (*r*_s_=0.52, *P*<0.01) (Fig. 4b).

The natural variation in photosynthesis within the cultivars was further explored using PCA. This uses a multi-dimensional dataset and reduces the dimensions into the smallest number of components that account for the most variation. Eleven photosynthetic parameters measured from all 64 cultivars were included in the PCA, which showed that 81% of the observed variation could be explained by four principal components (Table 2). Each principal component (PC) accounted for a proportion of the variation and was correlated to different degrees to the measured parameters. The first PC (PC1) accounted for the majority of the variation (33.6%), while PC4 accounted for the least at around 10.2%. Photosynthetic parameters that correlated with each PC are indicated as a heat map in Table 2. Briefly, *A*_max_ and *J*_max_ correlated most with PC1, *g*_m_ and Rubisco with PC2, *C*_i_^400^ and *A*_400_ with PC3, and *R*_d_ with PC4.

**Table 2.** Correlations of photosynthetic parameters with each principal component (PC)Strongest correlations are indicated in red, with weakest correlations as white. Standard deviation, proportion of variance explained, and cumulative proportion of variance explained for each PC are given below. *A*_max_, maximum photosynthetic CO_2_ assimilation; *J*_max_, maximum rate of electron transport demand for RuBP regeneration; *V*_cmax,_ maximum velocity of Rubisco for carboxylation; Carboxylation efficiency, the *in vitro* carboxylation efficiency of Rubisco; Rubisco content, Rubisco content of the flag leaf; *V*_c_^Rubisco^, velocity of Rubisco for carboxylation *in vitro*; *A*_400_, CO_2_ assimilation at ambient CO_2_ concentration (400 µmol mol^–1^ CO_2_); *R*_d_, day respiration rate; *g*_m_, mesophyll conductance for CO_2_ diffusion; *C*_i_^400^, internal CO_2_ concentration at ambient CO_2_ concentration (400 µmol mol^–1^ CO_2_); *C*_i_^max^, internal CO_2_ concentration at maximum photosynthetic CO_2_ assimilation.

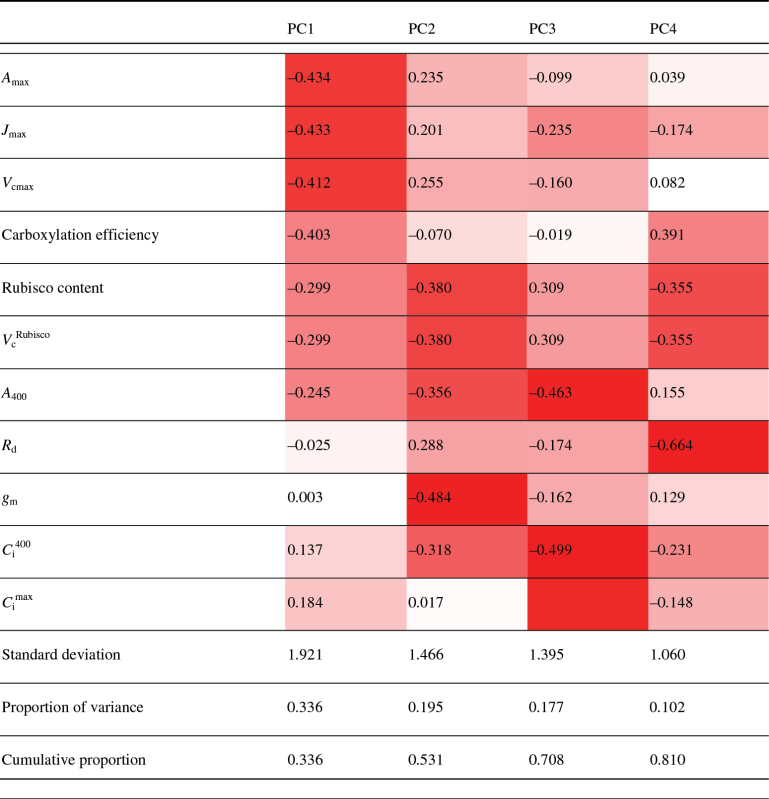

**Fig. 5.**
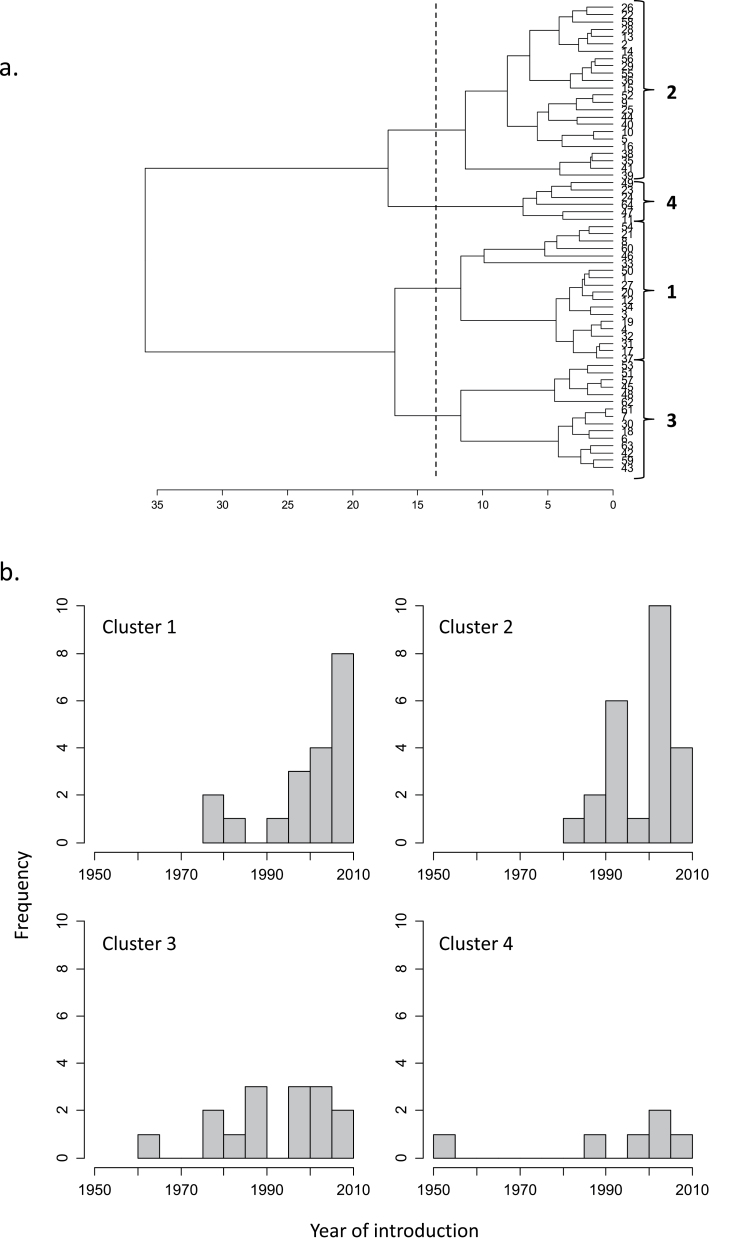
(a) Hierarchical clustering of cultivars for photosynthetic parameters, based on Euclidian distances. (b) Histograms of frequency distribution of year of introduction for cultivars per cluster.

Based on the PCA and accounting for a large proportion of the observed variation, hierarchical clustering was performed, yielding four groups (clusters) with common overall sources of variation, although different sources of variation were expected between clusters (Fig. 5a). Clusters 1 and 2 predominately consisted of modern cultivars, while clusters 3 and 4 contained the oldest cultivars 62 (Maris Widgeon, 1964) and 47 (Cappelle Deprez, 1953), as well as several more recently introduced varieties (Fig. 5b).

**Fig. 6.**
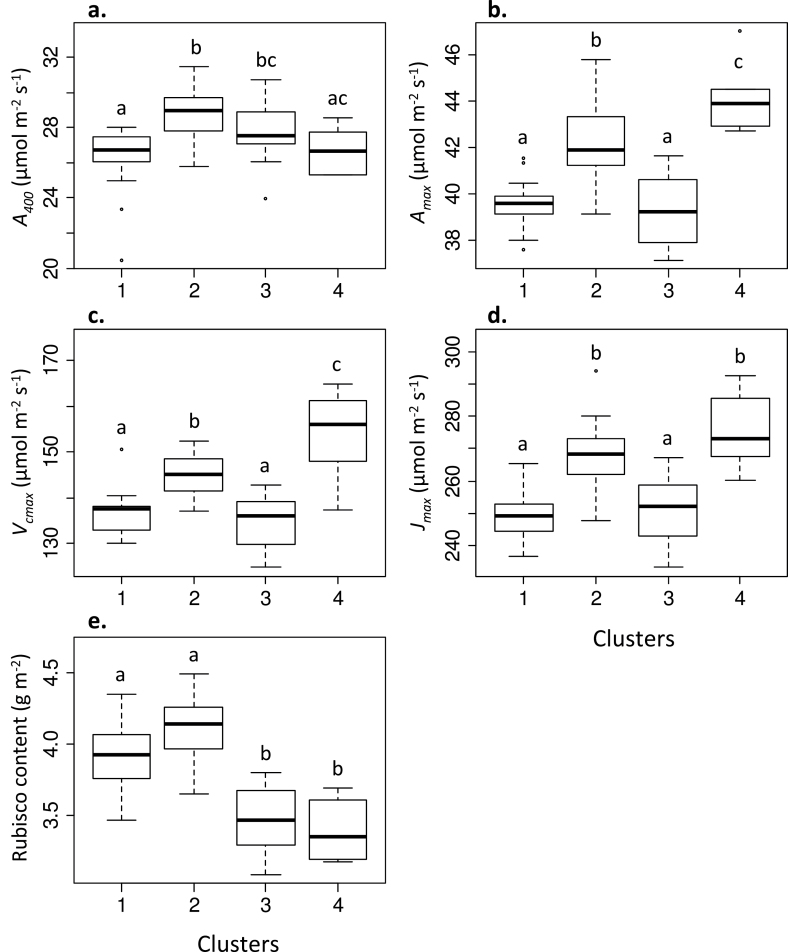
Means and variation for four clusters of (a) operational assimilation rate (*A*_400_), (b) maximum velocity of Rubisco carboxylation (*V*_cmax_), (c) maximum velocity of Rubisco carboxylation (*V*_cmax_), (d) the maximum rate of electron transport demand for RuBP regeneration (*J*_max_), and (e) Leaf Rubisco content. Significant differences are indicated (*P*<0.05)

Several significant differences in photosynthetic parameters among clusters were identified. Significant differences for *A*_400_ were observed between clusters 2 and 3, while mean values for clusters 1 and 4 did not differ (Fig. 6a). Clusters 2 and 4 had significantly higher mean values for *A*_max_, *V*_cmax_, and *J*_max_ compared with clusters 1 and 3 (Fig. 6b–d). Rubisco content was significantly greater in the cultivars of clusters 1 and 2 compared with clusters 3 and 4 (Fig. 6e). It is noteworthy that the highest *V*_cmax_ was found within cluster 4, although this cluster also contained the cultivars with the lowest amount of Rubisco. The opposite situation was found for cluster 1, which contained the cultivars with the lowest *V*_cmax_ and with the greatest amount of Rubisco. This indicates a relatively minor contribution of Rubisco compared with other traits under the prevailing conditions.

## Discussion

The products of photosynthesis are the primary determinants of plant productivity, and increasing photosynthesis has been widely recognized as a key trait to increase yields (Long *et al.*, 2006; Zhu *et al.*, 2010; Parry *et al.*, 2011; Raines, 2011). While biomass is a function of the total photosynthesis of the canopy over time, the flag leaves have, in the UK, been identified as the major contributor to grain yield (Thorne, 1973). Our analysis of flag leaf photosynthesis of 64 wheat cultivars revealed large variation in photosynthetic parameters as well as in yield and biomass and related traits. This is, to our knowledge, the largest systematic study of photosynthetic gas-exchange and agronomic parameters conducted on field-grown wheat to date. Although natural variation in photosynthetic capacity is known to exist among species (Wullschleger, 1993; Wright *et al.*, 2005; Hikosaka and Shigeno, 2009; Hikosaka, 2010; Lawson *et al.*, 2012) relatively few studies have examined natural variation either within species (Flood *et al.*, 2011) or in crop species (Pettigrew, 2004; Gilbert *et al.*, 2011; Gu *et al.*, 2014).

In this study, no consistent correlation was found between pre-anthesis flag leaf photosynthetic capacity and either grain yield or biomass when all cultivars were compared. Cultivars with the highest photosynthetic performance did not equate with the highest yields. Previous studies, using a range of different cultivars, have observed relationships between photosynthesis and yield (Fischer *et al.*, 1981; Blum, 1990; Fischer *et al.*, 1998; Reynolds *et al.*, 2000), while others have not (Chytyk *et al.*, 2011; Sadras *et al.*, 2012) or have refrained from drawing a definitive conclusion (Watanabe *et al.*, 1994). Most previous studies have demonstrated a positive relationship between photosynthesis and crop yield when measurements of operational or maximum photosynthesis rates were performed on flag leaves, at the time of grain filling under high-light conditions (Blum, 1990; Fischer *et al.*, 1998; Reynolds *et al.*, 2000; Furbank *et al.*, 2013). It is perhaps not surprising that we were unable to directly correlate photosynthetic capacity with yield, given that the measurements presented here were taken under conditions of saturating light, optimal to high CO_2_ concentrations, and without any stomatal limitation. In the field environment, even on days of full sunlight, conditions are rarely optimal and leaves will experience sun and shade flecks across the canopy due to changes in cloud cover, sun angle, self-shading, and shading from neighbouring plants (Way and Pearcy, 2012), and wind-driven movements (Lawson *et al.*, 2010). In this naturally fluctuating environment, stomata and photosynthesis respond continually to changing environmental cues, especially light and temperature and therefore lags in stomatal behaviour can limit photosynthesis through restricted CO_2_ diffusion (Lawson *et al.*, 1998; Lawson *et al.*, 2010; Lawson & Blatt, 2014). On top of these fluctuations in light, there are alterations in water status and local differences in humidity that will influence stomatal behaviour on short (minutes) and long (daily) timescales. This means that even instantaneous ‘snapshot’ measurements of gas exchange in the field rarely represent the average values achieved by the plant over a longer time period, and almost certainly will not provide a cumulative rate of photosynthesis over the season unless a large number of samples are obtained on different leaves and under all weather conditions covering diurnal and temporal variation within the canopy. Although no correlation could be drawn between photosynthetic capacity of the flag leaf and yield, we demonstrated considerable variability in capacity (33%) and growth parameters (including yield), illustrating the potential to exploit natural variation in existing wheat lines to improve photosynthesis in addition to the traits already selected.

Another interesting finding was that, although there were some large differences in *V*_cmax_ among clusters, comparable ranges of Rubisco content were found. For example, compare *V*_cmax_ and Rubisco ranges in clusters 1 and 2 and clusters 3 and 4 (see Fig. 6). Carboxylation efficiency of the measured flag leaves cannot be explained by Rubisco content, as the two parameters were only weakly correlated (correlation=0.12). This implies that other factors, for example Rubisco activase activity, determines carboxylation efficiency, as demonstrated by the significant correlation between functional Rubisco content and carboxylation efficiency (correlation= 0.36, *P*<0.01). This gives rise to the contention that the content of Rubisco, which typically accounts for 50% of soluble leaf protein (Ishimaru *et al.*, 2001), could potentially be reduced to benefit investment of nitrogen into other Calvin cycle enzymes and increase photosynthesis in this way, as has been proposed previously (Zhu *et al.*, 2010; Parry *et al.*, 2011). Increased light-saturated leaf CO_2_ assimilation rate (*A*_sat_) has been observed with a reduction in Rubisco content and a redistribution of nitrogen from Rubisco towards RuBP regeneration in both rice and wheat genotypes grown at elevated CO_2_ concentration (Makino *et al.*, 1997; Aranjuelo *et al.*, 2013). Similarly, high levels of variation have also been observed in Rubisco content, *A*_*max*_, stomatal conductance, and total leaf protein content in 10 rice varieties grown under identical conditions, some of which correlated with harvest index (Hubbart *et al.*, 2007). In the current study, however, as indicated in Table 3, the critical period between April and August 2012 was characterized by unusually low daytime irradiances (between 11 and 53% lower than the monthly 30-year averages) owing in large part to the uncharacteristically high rainfall (over 79% higher than the 30-year averages in April, June, and July). The extent to which Rubisco is limiting to photosynthesis depends largely on irradiance, being of greatest significance at high irradiances, with diminishing influence at lower irradiances (Stitt and Schulze, 1994). Owing to the uncharacteristically low irradiance over the growing season, a significant proportion of the Rubisco is likely to have been functionally redundant. Comparison of *V*_cmax_ predicted from direct measurement of Rubisco (by applying the rate constant for wheat Rubisco of Carmo-Silva *et al.*, 2010) with that derived from our photosynthesis measurements supports this notion, as the values based on the measured Rubisco content (mean *V*_cmax_=163±15 μmol m^–2^ s^–1^) were consistently higher than those derived from our photosynthesis measurements (mean *V*_cmax_=141±8 μmol m^–2^ s^–1^). It seems likely that, with higher ambient field irradiances, these alternative estimates would converge. Furthermore, canopy cover was larger under these field conditions than is normally found for many cultivars. This is likely to be due to the lower amount of sunshine but abundant rainfall received in the most important part of the growing season, which together facilitated increased canopy cover. This in turn would increase light interception, compensating for lower photosynthetic rates per unit leaf area.

**Table 3.** Monthly averages of sunshine, air temperature and rainfall for the growing season 2012Deviations from 30-year averages are shown in parentheses.

Month	Sunshine			Mean temperatures (°C)				Rainfall Total	
	MJ m^–2^ d^–1^	Hours	(%)	Max		Min		mm	(%)
January	2.8	82.2	(+20)	8.5	(+1.8)	2.5	(+1.3)	58.0	(–12)
February	5.3	109.3	(+29)	6.4	(–0.1)	0.1	(–0.8)	24.7	(–25)
March	10.2	193.5	(+79)	12.8	(+2.9)	3.1	(+0.4)	34.7	(–16)
April	12.2	150.1	(–11)	11.5	(–1.1)	3.3	(–0.7)	168.6	(+114)
May	15.3	175.6	(–19)	16.1	(+0.1)	7.9	(+1.0)	52.6	(–2)
June	15.2	144.9	(–53)	17.6	(–1.5)	10.0	(+0.3)	166.5	(+113)
July	15.6	172.3	(–33)	19.8	(–2.0)	11.6	(–0.2)	128.4	(+79)
August	14.3	176.5	(–20)	21.7	(+0.2)	12.8	(+0.9)	54.9	(–9)
September	11.8	179.6	(+36)	18.3	(+0.0)	8.4	(–1.5)	40.4	(–17)
October	5.8	86.0	(–26)	12.7	(–1.4)	6.7	(–0.5)	115.8	(+34)
November	3.2	76.9	(+6)	9.4	(–0.3)	3.6	(–0.2)	100.4	(+24)
December	2.1	68.2	(+14)	7.5	(+0.6)	1.4	(–0.2)	114.2	(+45)

There are arguments that natural selection has already maximized photosynthesis and that further manipulation of photosynthesis would not lead to further gains. However, as highlighted by Leister (2012), natural selection has not selected for agronomic yield but has maximized plant fitness for survival in an environment vastly different to our ‘resource- rich’ arable fields. Figures 5 and 6 of this study also illustrate that previous breeding strategies may have unintentionally selected for traits other than those associated with photosynthesis. From the cluster analysis, we can see that the clusters that contained the highest *V*_cmax_ values (Fig. 6c, cluster 4) contained some of the oldest varieties (Fig. 5d), while low values of *A*_max_, *V*_cmax_, and *J*_max_ (Fig. 6, clusters 1 and 3) were made up of mainly modern varieties (Fig. 5, cluster 1) and a broad range of recent and early introduced cultivars (Fig. 5, cluster 3). The cluster analysis strongly suggests that selective breeding programmes have unintentionally selected for cultivars with low capacities. The aim of this study was to quantify the degree of natural variation in existing wheat cultivars with the expectation that such information could be incorporated into future breeding programmes to aid in the selection of traits associated with photosynthetic capacity and performance. The question remains as to how the observed natural variation in photosynthetic capacity could be exploited to improve photosynthetic performance and yield. Conventional breeding approaches could be applied for selection of, for example, higher *J*_max_, but the heritability for this particular trait in the current study was not very high (*h* ^2^=0.32). However, large-scale phenotyping approaches for photosynthetic traits, such as used in the current study, are the first step towards the genetic dissection of these traits. It is widely accepted that phenotyping to discover dependable levels of expression for traits and associated genetic markers can facilitate their use in breeding (Reynolds *et al*., 2009, 2012b; Rebetzke *et al.*, 2013). Marker-assisted selection for these and other photosynthetic traits should be possible, as the wheat genome has been sequenced and gene families associated with crop productivity are being identified (Brenchley *et al.*, 2012). Moreover, varietal single-nucleotide polymorphisms are now available for over half of the 64 cultivars used in this study and are freely accessible (Wilkinson *et al.*, 2012). This information will assist the breeding for improved photosynthetic performance in wheat. Although such improvements may be difficult to achieve through conventional breeding, genetic manipulation of RuBP regeneration capacity has been accomplished in tobacco by increasing the levels of sedoheptulose-1,7-biphospatase (SBPase), which has been shown to increase *J*_max_, photosynthetic performance, and increased plant growth and yield (Lefebvre *et al.*, 2005). These tobacco lines demonstrated that modest differences in the CO_2_ dependence of photosynthesis (which were considerably smaller than the differences highlighted in the *A*/*C*_i_ responses of Fig. 2) can translate into very significant differences in growth over the lifetime of the plant. Mathematical modelling and numerical simulations have been used to identify several enzymes, including SBPase, as being potentially limiting to photosynthesis (Poolman *et al.*, 2000; Zhu *et al.*, 2007).

Whether achieved through the selection of naturally occurring photosynthetic genetic markers or by means of genetic manipulation, the extent to which improved photosynthetic potential impacts on yield will depend upon prevailing abiotic and biotic environmental conditions, together with the genetic background of the cultivars in question (which will influence the ability to thrive in suboptimal environments, including variations/extremes of temperature, water, sunlight, nutrient availability, herbivory, and/or fungal pathogens). Yield is an important criterion in the selection of all commercial wheat cultivars, although other performance-related characteristics have also been represented, to differing extents, in the chosen genotypes. Apart from major genes that control photoperiod sensitivity (*Ppd*) and height (*Rht*) and are directly related to yield, several resistance genes were present among the chosen cultivars. Of the leaf rust resistance genes, *Lr37* is found in 18 recent cultivars (earliest year of introduction 1997) and *Pch1* is found in only five modern cultivars (earliest year of introduction 2005). In the former group, half of the cultivars were assigned to cluster 1, and of the latter group, cultivars were only present in clusters 1 and 2. These clusters showed a generally lower photosynthetic performance. Although these resistance genes clearly have an advantage in the protection against pathogens, they may not benefit the photosynthetic performance of the plant.

However, as the example of SBPase overexpression (above) illustrates, when discrete, appropriate and specific genetic changes are made to plants growing under similar conditions, very clear yield benefits can accrue. As highlighted by Lawson *et al.* (2012), potential maximum photosynthetic capacity is rarely achieved in the field even under favourable conditions. Such observations can be explained by stomatal limitation due to limited water availability and to a lag in stomatal behaviour relative to changes in photosynthesis under fluctuating environmental (mostly PPFD) conditions. Additionally, factors such as defence against biotic and abiotic stress and nitrogen availability and distribution may play a role, for example if the expression of resistance genes were to impact negatively on photosynthetic performance. To assess these limitations in terms of potential versus operational photosynthetic capacity, further measurements under field and controlled conditions are necessary.

Using a simulation analysis, Gu *et al.* (2014) determined the contribution of natural variation in photosynthetic rate (*A*) to productivity in rice. Genetic variation (25%) in both Rubisco-limited and electron transport-limited photosynthesis increased rice yields by 22–29% across different locations and years. This illustrates that rice production could be significantly improved by exploiting existing variation in germplasm (Gu *et al.*, 2014). Variation in photosynthetic capacity and stomatal conductance in soybean genotypes has also been reported (Gilbert *et al.*, 2011), which resulted in differences in intrinsic water-use efficiency, leading to the suggestions that a breeding strategy could be employed to produce ‘water-saving soybeans with high photosynthetic capacities’, that would ultimately benefit crop yields. These studies and others illustrate the potential of exploiting natural variation in photosynthesis as an approach to increasing crop yield, and similar increases in crop yield are expected for wheat.

The current study is, to our knowledge, the largest study of photosynthetic gas-exchange and growth parameters for wheat to date, and demonstrates significant natural variation in photosynthetic capacity, growth, and yield between existing wheat cultivars, providing an invaluable resource for the improvement of photosynthetic capacity and yield in wheat.

## Supplementary data

Supplementary data are available at *JXB* online.

Supplementary Fig. S1. Frequency distribution (histogram) of cultivars for years of introduction as used in the current study.

Supplementary Fig. S2. Development of cultivars over time (Zadoks scale).

Supplementary Fig. S3. Meta-analysis of all cultivars for relationships between measured parameters with correlation and significance, frequency distribution per parameter and regression plots.

Supplementary Table S1. Daily mean light levels during the period of photosynthetic measurements.

Supplementary Data

## Abbreviations:

*A*: CO_2_ assimilation
ANOVA: analysis of variance
CABP: 2′-carboxyarabinitol-1, 5-bisphosphate; *C*_i_, intercellular CO_2_ concentration; *C*_i_, intercellular CO_2_ concentration
ERYCC: Earliness & Resilience for Yield in a Changed Climate
g_m_: mesophyll conductance
*h*^2^: heritability
*J*_max_: maximum rate of electron transport demand for RuBP regeneration
PC: principle component
PCA: principle components analysis
PEG: polyethylene glycol
PPFD: photosynthetic photon flux density
*R*_d_: respiration rate
Rubisco: ribulose-1,5-bisphosphate carboxylase/oxygenase
RuBP: ribulose-1,5-bisphosphate
SBPase: sedoheptulose-1,7-biphospatase
*V*_cmax_: maximum velocity of Rubisco for carboxylation.
