# Supplementary material for: Natural variation in photosynthetic capacity, growth, and yield in 64 field-grown wheat genotypes
Source: J Exp Bot. 2014 Jun 24;65(17):4959–73. doi: 10.1093/jxb/eru253 (PMC4144772; doi:10.1093/jxb/eru253)
Supplement: Supplementary Data [file supp_65_17_4959__index.html]

Natural variation in photosynthetic capacity, growth, and yield in 64 field-grown wheat genotypes — Supplementary Data 

# Natural variation in photosynthetic capacity, growth, and yield in 64 field-grown wheat genotypes

## Supplementary Data

Data files

**Files in this Data Supplement:**

- Supplementary Data - Supplementary Data
