# Supplementary material for: Natural variation in photosynthetic capacity, growth, and yield in 64 field-grown wheat genotypes
Source: J Exp Bot. 2014 Jun 24;65(17):4959–73. doi: 10.1093/jxb/eru253 (PMC4144772; doi:10.1093/jxb/eru253)
Supplement: Supplementary Data [file supp_eru253_Driever_et_al_Suppl_figures_and_tables.pdf]

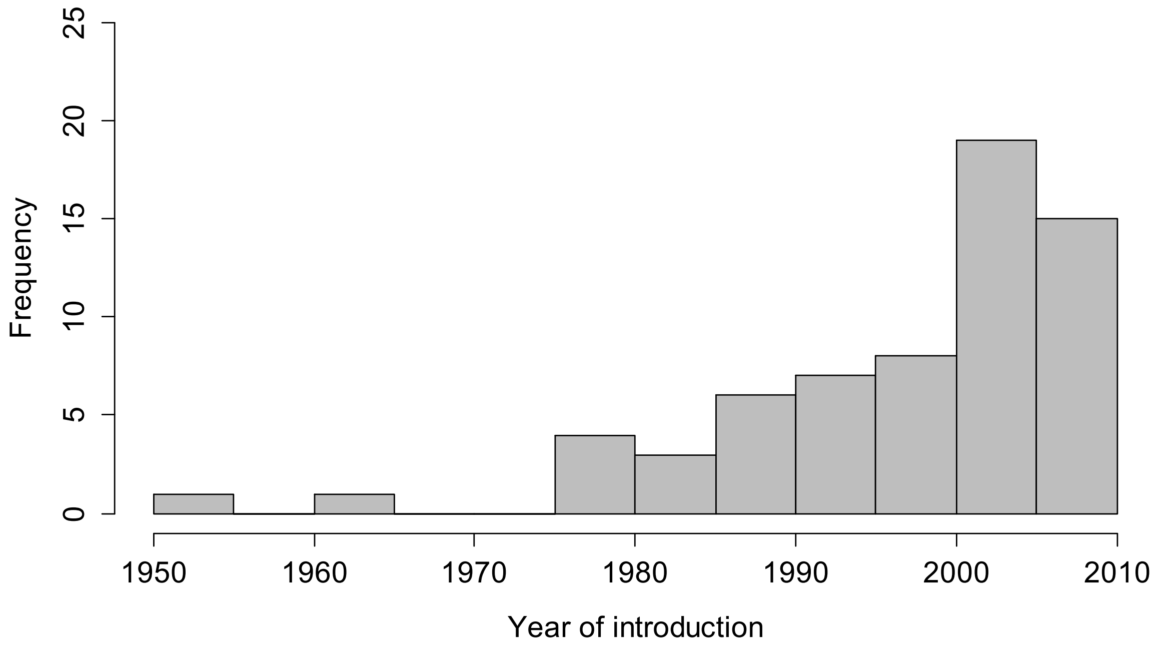

Suppl. Figure 1. Frequency distribution (histogram) of cultivars for years of introduction as used in the current study.

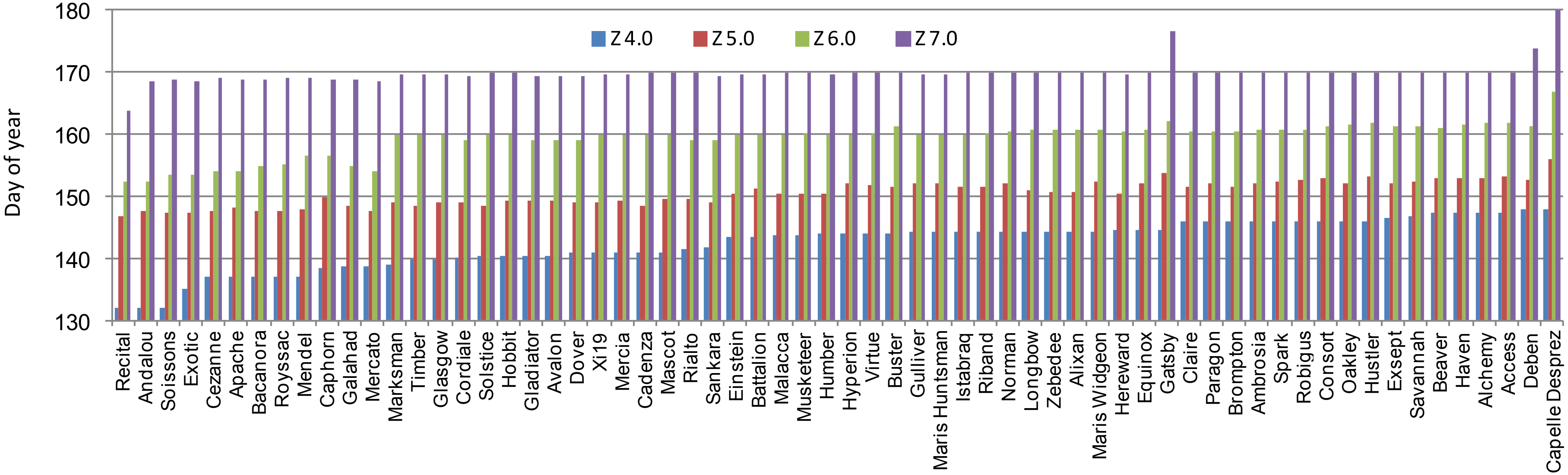

Suppl. Figure 2. Development of cultivars over time (Zadoks scale). The times taken for each variety to reach key growth stages are indicated. 4.0 = flag leaf fully emerged/start of booting; 5.0 = start of ear emergence; 6.0 = start of anthesis; 7.0 = start of milk development in kernel.

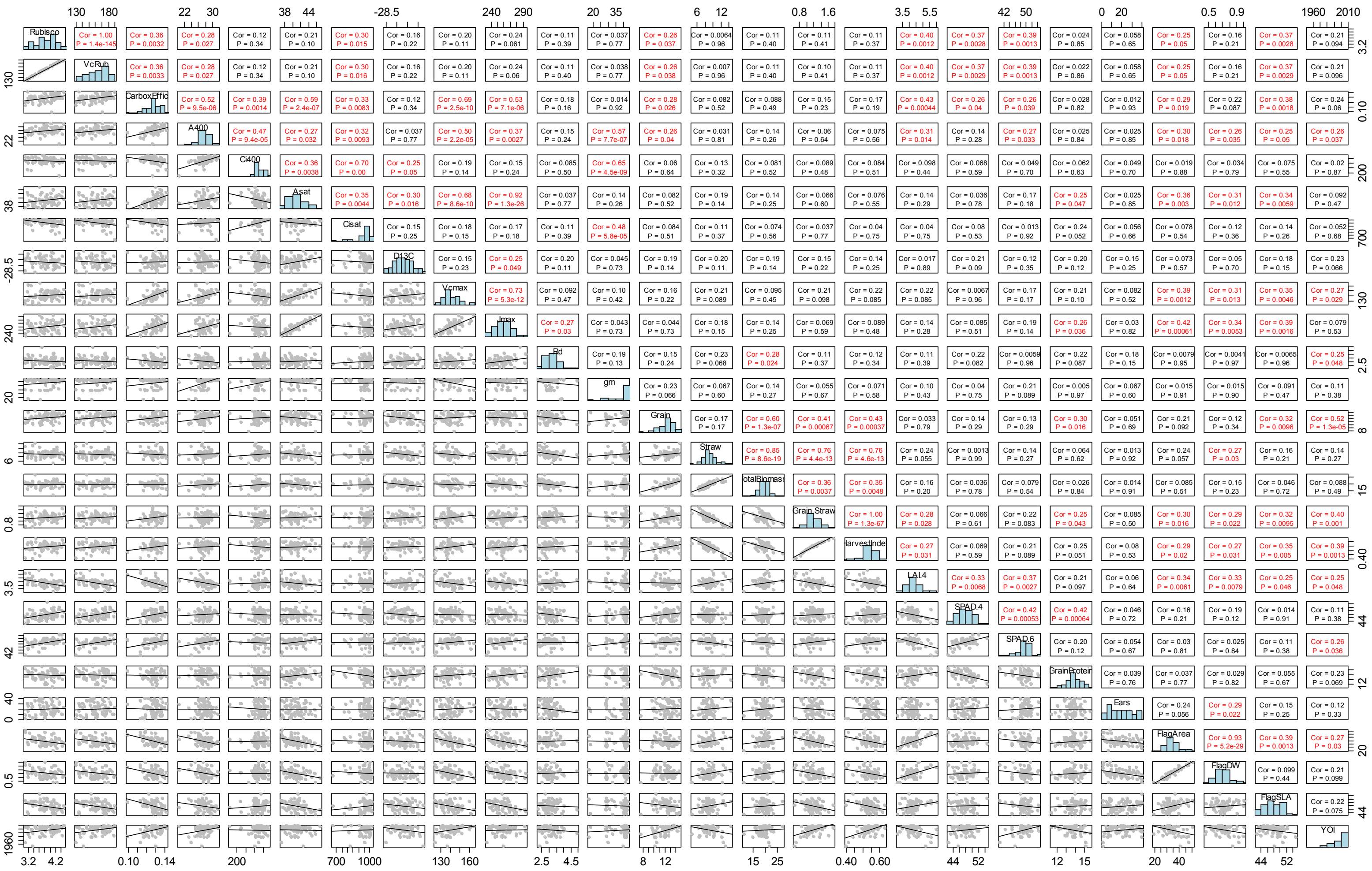

Suppl. Figure 3. Meta-analysis of all cultivars for relationships between measured parameters with correlation and significance (upper panels), frequency distribution per parameter (histograms, diagonal panels) and regression plots (lower panels). Names of measured parameters are given in the histogram titles.

Suppl. Table 1. Daily mean light levels during the period of photosynthetic measurements

Day of year	Daily mean solar PAR ($\mu mol\ m^{-2}\ s^{-1}$)
132	77.2
133	257.6
134	208.8
135	246.5
136	82.6
137	113.7
138	249.6
139	197.6
140	99.1
141	188.1
142	67.4
143	137.1
144	224.2
145	215.3
146	198.25
147	330.9
148	332.25
